# Neonatal Gastric Lactobezoar: Management with N-Acetylcysteine

**DOI:** 10.1155/2012/412412

**Published:** 2012-12-02

**Authors:** Sarah Bajorek, Roel Basaldua, Katherine McGoogan, Charla Miller, Craig B. Sussman

**Affiliations:** ^1^Department of Pediatrics, University of Florida, Jacksonville, FL 32209, USA; ^2^Division of Neonatology, Wolfson Children's Hospital, Jacksonville, FL 32209, USA; ^3^Division of Pediatric Gastroenterology, Nemours Children's Clinic, Jacksonville, FL 32209, USA

## Abstract

Gastric lactobezoars (GLBs) are the most common form of bezoars in neonates and consist of aggregations of undigested milk constituents. GLB can present with a variety of intra-abdominal clinical symptoms, and occasionally, extra-abdominal symptoms. Conservative management, with a period of bowel rest and intravenous fluids, is the most common treatment regimen for uncomplicated GLB. Surgical measures are reserved for the rare complications of obstruction and/or perforation. Although limited, utilization of the protein-cleaving enzyme N-acetylcysteine has been described for the disintegration of GLB in toddlers. In this paper, we discuss the first documented use of N-acetylcysteine for a neonatal GLB. Supporting literature, the infant's unusual presentation, and details of the treatment regimen are discussed.

## 1. Introduction

A bezoar is an exogenous mass trapped in the gastrointestinal system, usually made of proteinaceous material. Gastric lactobezoars (GLBs) are the most common form in neonates and consist of aggregations of undigested milk constituents [1]. GLB can present with a myriad of nonspecific symptoms making the diagnosis challenging. In the minority of cases, a GLB can manifest itself as a gastric obstruction and/or perforation necessitating surgical measures [2]. Further, the management of GLB remain controversial with the mainstay of therapy consisting of bowel rest and parenteral nutrition [2]. 

 In a recent case series, intragastric use of the protein-cleaving enzyme N-acetylcysteine (NAC) for the disintegration of GLB in toddlers was successful [3]. NAC possesses a number of clinical uses in the neonatal population including enemas for meconium ileus, intravenous use in hemochromatosis, topical application for ichthyosis, and as a pulmonary inhalant for respiratory disorders such as meconium aspiration syndrome (MAS) [4–7]. To date, the use of NAC has not been documented as a method for GLB disintegration in neonates. In the following case report, we present a relatively rare presentation of a GLB. Further, we discuss the first documented disintegration of a GLB utilizing NAC in a neonate.

## 2. Case Report

The patient was a term male infant born via vaginal delivery complicated by meconium stained amniotic fluid. The infant was vigorous at delivery and received APGAR scores of 8 and 8. Shortly after birth he developed respiratory distress with hypoxemia. The infant subsequently suffered from MAS and persistent pulmonary hypertension. He required maximum support including high frequency ventilation, blood pressure augmentation, inhaled nitric oxide, and eventually veno-venous extracorporeal membrane oxygenation (ECMO) for 5 days. 

**Figure 1:**
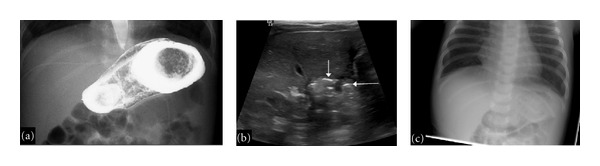
Radiographic findings of a neonatal gastric lactobezoar. (a) Upper GI demonstrating a large filling defect outlining the lumen of the stomach. (b) Sagittal ultrasound of the abdomen showing highly echogenic intrabezoaric air trapping (arrows) within a low echogenic lactobezoar. (c) AP chest X-ray with suggestive findings of a GLB. Intraluminal air outlines an opaque intragastric mass.

Once stable, enteral feedings of term cow's milk formula were introduced, and by day of life 18, the infant was tolerating full volume feeds. Due to poor weight gain he was changed to 24 kcal/oz formula. He remained intermittently tachypneic with occasional episodes of wheezing. Fluid restriction was enforced and he received loop diuretics for 18 days. He continued to have episodes of wheezing that inconsistently responded to inhaled *B*-2 agonists and oral steroids. Due to poor oral-motor skills and wheezing episodes, he underwent a contrast swallow study and upper GI to evaluate for micro-aspiration. No micro-aspiration was observed; however, a significant gastric filling defect was identified consistent with a GLB (Figure 1(a)). To further investigate, an abdominal ultrasound demonstrated echogenic material within the gastric lumen (Figure 1(b)). In review of a prior chest X-ray, findings of a GLB were suggestive (Figure 1(c)). The infant appeared to be clinically void of any gastrointestinal symptoms, except that his oral intake the week prior had gradually decreased.

Based on the literature in toddlers, we used 10 mg/kg/dose of 10% NAC diluted with 50 mLs of normal saline for intragastric disintegration [3]. This was administered via nasogastric tube (NGT) over thirty minutes followed by clamping of the NGT for two hours. Stomach contents were aspirated at three and six hours post administration. This regimen was repeated every six hours until aspiration did not yield any evidence of a GLB. Seven total doses of NAC were required over forty-eight hours. Once the stomach was void of any particulate matter, the infant was restarted on oral feeds of Enfalyte (Mead Johnson, Evansville, IN). A repeat abdominal ultrasound documented resolution of the GLB. The infant was transitioned to a term 20 kcal/oz, lactose-free, hydrolyzed hypoallergenic formula without complications. The decision to trial a hypoallergenic formula was based on the findings of recurrent wheezing of an unclear etiology, and not due to the GLB. The infant underwent a complete infectious, pulmonary, and otolaryngotomy evaluation without identification of an underlying cause. His feeding volumes increased and respiratory symptoms improved as discussed below. 

## 3. Discussion

Gastric lactobezoars (GLBs) are the most common type of bezoars found in infants [1]. They were first described and radiographically depicted in low birth weight infants in 1959 [8]. In that report, swallowed contrast was utilized to illustrate the GLB, with the subsequent treatment consisting of gastrotomy [8]. The pathogenesis of GLB includes mostly exogenous influences such formulas with high casein contents, medium chain triglyceride oils, and increased caloric density milk. Endogenous factors include an immature gastrointestinal tract and dehydration [2]. However, a GLB has been documented in a term, exclusively breastfed infant [9]. Our patient possessed some of the above risk factors including consuming a high caloric density formula in combination with fluid restriction and diuretic use. Clinicians must be cognizant that these clinical management decisions may result in the development of a GLB. 

GLB can present with a variety of clinical symptoms. Frequently, they manifest with abdominal distention, vomiting, diarrhea, or a palpable mass. Occasionally, respiratory or cardiovascular symptoms are observed [2]. GLB are under-diagnosed and often not included in the differential diagnosis for the above mentioned, non-specific symptoms. 

In this particular case, the infant's oral intake was diminishing. He did not display the well-documented gastrointestinal symptoms observed with GLB. However, the neonate had episodes of wheezing thought to be related to underlying MAS. Respiratory distress and apnea have been described in association with GLB [2]. Further, a single case of a nine month old who developed eczema, asthma, and cow's milk protein intolerance in combination with a GLB has been documented [10]. One may speculate that our patient had an allergic component to his respiratory symptoms. Once disintegration of the GLB was accomplished, and hypoallergenic formula initiated, he demonstrated complete resolution of wheezing. 

A trial of bowel rest and intravenous fluids, with or without normal saline gastric lavage, has become the preferred initial treatment method for GLB [2]. This conservative regimen appears to be successful in over eighty-five percent of treated cases [2]. In those cases where the GLB does not dissolve, or is complicated by obstruction and/or perforation, surgical management is indicated.

Although limited, intragastric use of NAC for the disintegration of GLB in toddlers and bezoars in adults has been previously described [3, 11]. NAC is indicated as a mucolytic adjunctive therapy for abnormal, viscid, and inspissated mucus secretions. The mechanism of action is via the breakage of disulfide linkages in mucus, thereby lowering viscosity [12]. This same mechanism likely attributes to its ability to break down mucopolysaccharide fibers in GLB [3]. Although it was speculated that the therapeutic effect was related to NAC, the possibility of the GLB disintegration with the normal saline diluent alone cannot be excluded. The most common adverse effects associated with oral use of NAC are nausea and vomiting [13]. Of note, temporary hepatic derangement following NAC enemas in infant's with cystic fibrosis has been reported [14]. Hepatic enzymes were not monitored in our patient; however this should be considered for future uses, especially if the regimen is prolonged. 

GLB may be one of the great impersonators of neonatal diseases based on the array of non-specific symptoms they exhibit. The clinician must hold a high index of suspicion to seek out a GLB. When discovered, the management strategy remains ambiguous and must be tailored to the individual neonate. A conservative approach is likely the safest and most appropriate for a preterm infant with an immature GI tract, while aggressive surgical management is required for perforations and some obstructions regardless of gestation age. In this case presentation, the infant described was full term and clinically stable. Our goal was rapid disintegration of the GLB, while avoiding long-term intravenous access with parenteral nutrition and additional complications. We present this method as a reasonable treatment alternative for the term infant who presents with a nonsurgical GLB. Also, this case represents a potentially unique presentation of a GLB with concurrent cow's milk protein allergy. We therefore suggest that a GLB should be considered in the differential diagnosis for an infant with similar clinical findings. Controlled studies are recommended to determine the safety and efficacy of NAC use for this clinical scenario.

## Abbreviations

GLB: Gastric lactobezoars
ECMO: Extracorporeal membrane oxygenation
MAS: Meconium aspiration syndrome
NAC: N-acetylcysteine
NGT: Nasogastric tube.
